# Reduced adiposity attenuates FGF21 mediated metabolic improvements in the Siberian hamster

**DOI:** 10.1038/s41598-017-03607-x

**Published:** 2017-06-26

**Authors:** Jo E. Lewis, Ricardo J. Samms, Scott Cooper, Jeni C. Luckett, Alan C. Perkins, Andrew C. Adams, Kostas Tsintzas, Francis J. P. Ebling

**Affiliations:** 1 School of Life Sciences, University of Nottingham Medical School, Queen’s Medical Centre, Nottingham, NG7 2UH UK; 2 Lilly Research Laboratories, Indianapolis, IN 46285 USA; 3 Radiological Sciences, School of Medicine, University of Nottingham Medical School, Queen’s Medical Centre, Nottingham, NG7 2UH UK; 4 MRC/ARUK Centre for Musculoskeletal Ageing, School of Life Sciences, University of Nottingham Medical School, Queen’s Medical Centre, Nottingham, NG7 2UH UK

## Abstract

FGF21 exerts profound metabolic effects in Siberian hamsters exposed to long day (LD) photoperiods that increase appetite and adiposity, however these effects are attenuated in short day (SD) animals that display hypophagia and reduced adiposity. The aim of this study was to investigate whether the beneficial effects of a novel mimetic of FGF21 in the LD state are a consequence of increased adiposity or of the central photoperiodic state. This was achieved by investigating effects of FGF21 in aged hamsters, which is associated with reduced adiposity. In LD hamsters with increased adiposity, FGF21 lowered body weight as a result of both reduced daily food intake and increased caloric expenditure, driven by an increase in whole-body fat oxidation. However, in LD animals with reduced adiposity, the effect of FGF21 on body weight, caloric intake and fat oxidation were significantly attenuated or absent when compared to those with increased adiposity. These attenuated/absent effects were underpinned by the inability of FGF21 to increase the expression of key thermogenic genes in interscapular and visceral WAT. Our study demonstrates the efficacy of a novel FGF21 mimetic in hamsters, but reveals attenuated effects in the animal model where adiposity is reduced naturally independent of photoperiod.

## Introduction

Fibroblast growth factor 21 (FGF21) is a member of the endocrine family of FGFs that lack a heparin-binding domain, which allows for secretion into the circulation^1^. Whilst the primary source of FGF21 is the liver, the systemic effects of FGF21 are mediated by its binding to a complex composed of a receptor (FGFR1) and a co-factor β-klotho (KLB) in the central nervous system and adipose tissue^2–5^.

Pharmacological administration of FGF21 in obese mice improved insulin sensitivity and glycaemic control, reduced dyslipidaemia and promoted energy expenditure and weight loss, whilst treatment of non-human primates with recombinant human FGF21 or an FGF21 mimetic (LY2405319; LY) led to a dramatic and rapid lowering of body weight, glucose, insulin, cholesterol and triglyceride levels^3, 4, 6–9^. Furthermore, in individuals with type 2 diabetes mellitus (T2DM), chronic administration of an FGF21 analogue decreased body weight and improved plasma lipid profiles^10^.

Importantly, deleting either FGFR1 or KLB in adipose tissue, demonstrates that activation of this organ is intrinsically required for FGF21’s pharmacological effects^11, 12^. Similarly, adipose-specific over-expression of KLB sensitises mice to endogenous FGF21 action^13^. In addition to this, in response to cold stress, FGF21 is induced and then acts in an autocrine/paracrine manner to augment the activity of thermogenic pathways in both brown (BAT) and white adipose tissue (WAT)^2, 14–17^. Indeed, the protection against diet-induced obesity (DIO) observed in FGF21 transgenic mice is proposed to be a consequence of increased BAT mass and thermogenic activity^5, 18^. Whilst uncoupling protein 1 (Ucp1) is required for the full magnitude of the FGF21-induced increase in energy expenditure^19, 20^.

We have previously shown that the effects of exogenous FGF21 on appetite and fat oxidation (FOX) are attenuated in short day (SD) Siberian hamsters, a natural model of adiposity/leanness, but it is unclear whether this a reflects photoperiod-induced central change in response, or is due to the diminished adiposity in SD^21^. Our work utilising a monoclonal antibody (IMC-H7), which targets FGFR1c, suggests that the photoperiodic state is important in determining the response^22^. However, the actions of FGF21 additionally require the presence of KLB, therefore our studies with IMC-H7 may not directly inform our understanding of the factors which determine FGF21 responsiveness^3^. Therefore we intend to assess the efficacy of the FGF21 mimetic LY in the Siberian hamster and to determine whether the attenuated activity of FGF21 in SD hamsters is a result of reduced adiposity or central photoperiodic state, using a natural model of reduced adiposity whilst maintaining the central photoperiodic state^23, 24^. In our present study, we demonstrate the efficacy of LY, whilst demonstrating, for the first time, the attenuated effects of treatment in aged Siberian hamsters, where adiposity is reduced to SD levels, despite increased food intake and the maintenance of the LD phenotype.

## Results

### Ageing is associated with reduced adiposity in the Siberian hamster, a natural model of adiposity/leanness

Body weight was significantly reduced in the aged animals, despite significantly increased daily food intake (p < 0.05, Figure S1A and B). Analysis of body composition using computed tomography demonstrated a significant reduction in the volume of both interscapular and visceral white adipose tissue in aged animals (p < 0.05, Figure S1C). Neither energy expenditure nor ambulatory activity was different between the groups (Figure S1D and E), however, there was a trend towards increased RER (p = 0.06, Figure S1F), a likely consequence of increased food intake in the aged animals. Whilst circulating FGF21 did not differ between the groups (Figure S1G), leptin was significantly reduced in aged animals (Figure S1H, p < 0.05). Furthermore, expression of the FGF21 receptor complex (FGFR1c/KLB) in the hypothalamus and in adipose tissue (interscapular BAT ﻿(iBAT), interscapular WAT (iWAT) and perirenal﻿ WAT﻿ (prWAT) was unaffected by age in the Siberian hamster (Figure S2).

### Aged animals treated with FGF21 demonstrate attenuated effects on body weight and composition as well as food intake

**Figure 1:**
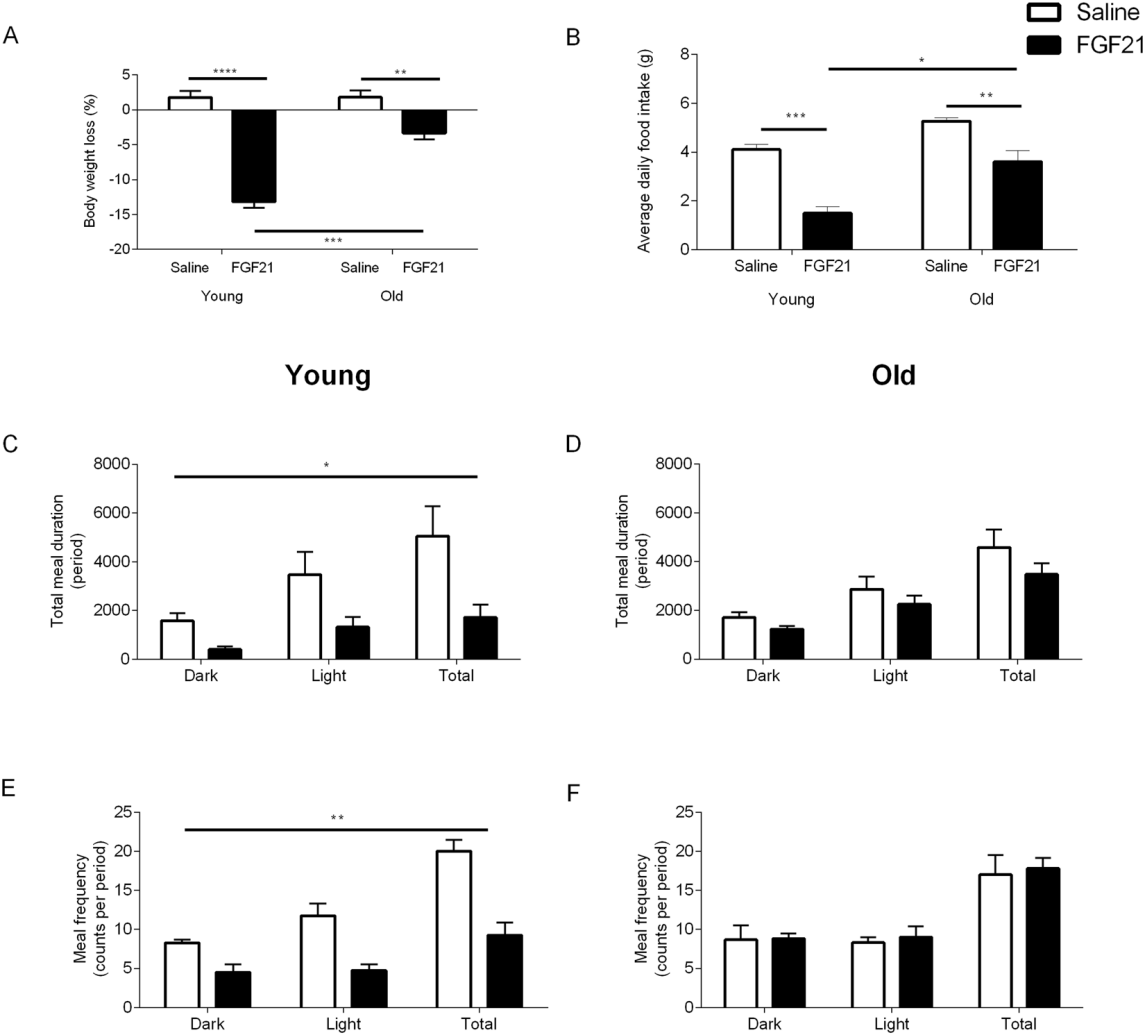
Reduced adiposity attenuates the effects of the FGF21 in the Siberian hamster. Percentage body weight loss (**A**), average daily food intake (**B**), meal duration (**C** and **D**) and meal frequency (**E** and **F**) of young and aged Siberian hamsters treated with vehicle (saline) or FGF21. Values are group mean ± SEM, n = 5–6 per treatment: *p < 0.05, **p < 0.01, ***p < 0.001, ****p < 0.0001.

Young and aged animals chronically treated with the FGF21 mimetic, LY, demonstrated a progressive decrease in body weight (Fig. 1A, p < 0.05). At the end of the 7 day treatment period, body weight was decreased by approximately 13% and 3% respectively in the young and aged cohorts of Siberian hamsters, as compared to the age- and weight-matched vehicle-treated controls. The weight loss was attenuated in the aged cohort of Siberian hamsters treated with FGF21 (p < 0.05, Fig. 1A). Similarly, daily food intake was significantly reduced by FGF21 treatment in young (p < 0.05) and aged (p < 0.05) cohorts by approximately 64 and 32% respectively compared to vehicle-treated control groups. Again, the reduction in food intake was significantly attenuated in the aged cohort of Siberian hamsters treated with FGF21 (p < 0.05, Fig. 1B). Meal duration and frequency was significantly reduced by treatment in the young cohort (p < 0.05, Fig. 1C and E), an effect which was not apparent in the aged cohort (Fig. 1D and F).

**Figure 2:**
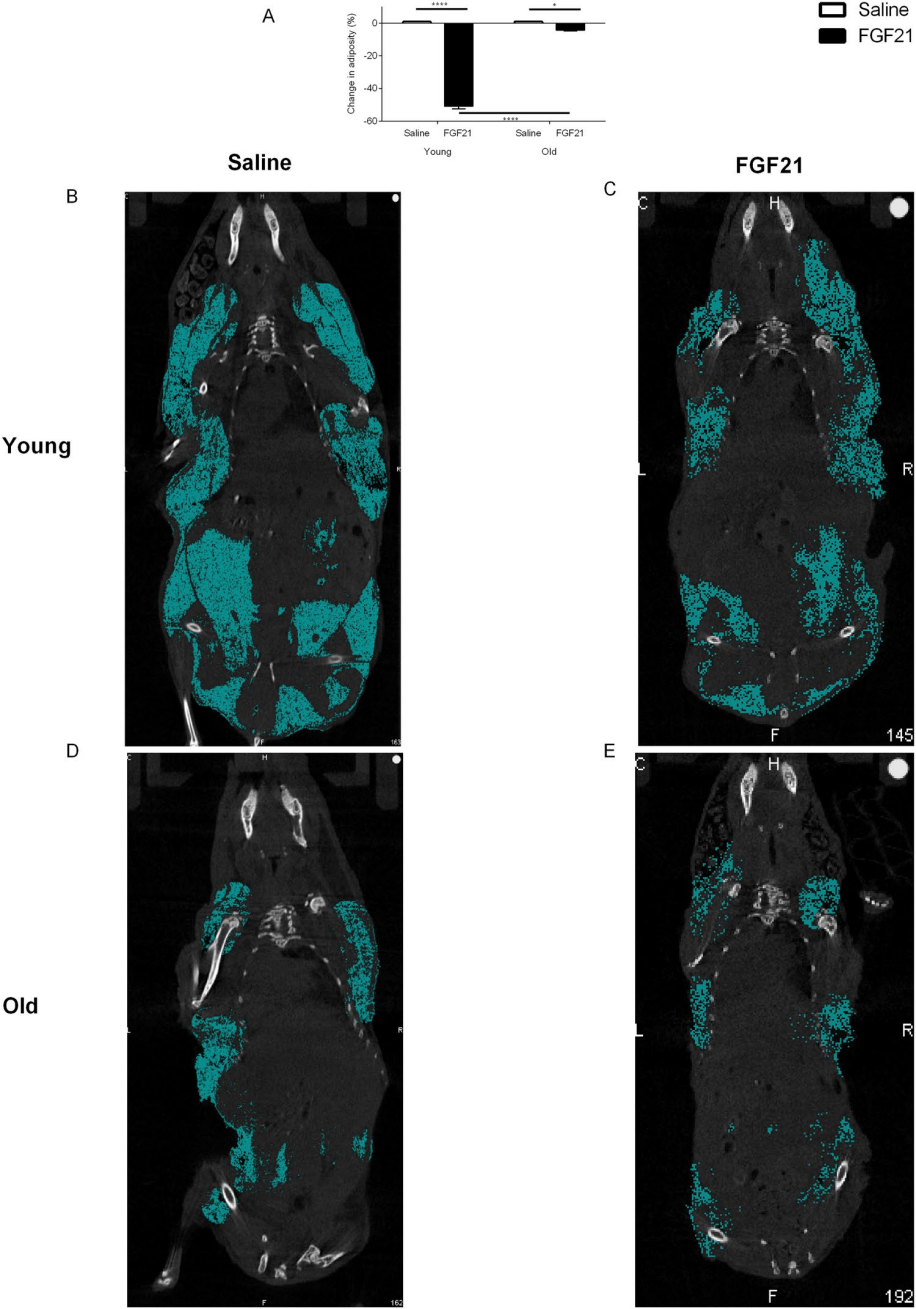
CT assessment demonstrates FGF21 reduces total adiposity in young and aged Siberian hamsters. Percentage change in total adiposity (**A**). Values are group mean ± SEM, n = 5–6 per treatment: *p < 0.05, **p < 0.01, ****p < 0.0001. Representative images of young (**B** and **C**) and aged (**D** and **E**) Siberian hamsters treated with vehicle (saline) or FGF21 respectively.

Body composition analysis demonstrated that in response to treatment, the volumetric change in interscapular and visceral adiposity was significantly reduced in both young and aged animals (Fig. 2A, p < 0.05). However, this effect of treatment was significantly attenuated in aged animals (Fig. 2A, p < 0.05).

### FGF21 increases energy expenditure in young and aged animals

**Figure 3:**
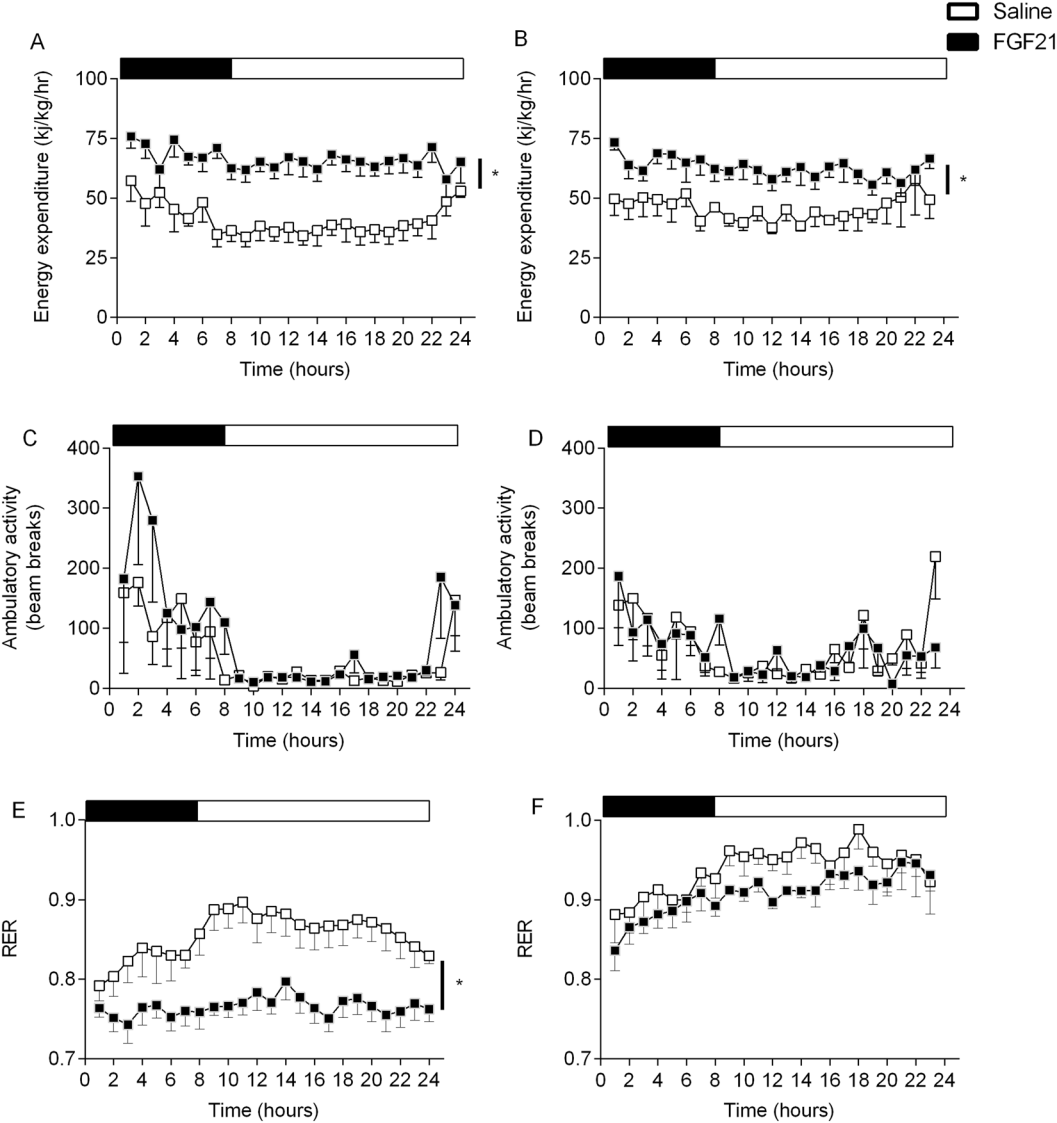
Treatment with FGF21 increases energy expenditure but fails to reduce RER in the aged cohort of Siberian hamsters. Energy expenditure (**A** and **B**), ambulatory activity (**C** and **D**) and RER (**E** and **F**) of young and aged Siberian hamsters treated with vehicle (saline) or FGF21. Values are group mean ± SEM, n = 5–6 per treatment: *p < 0.05.

**Figure 4:**
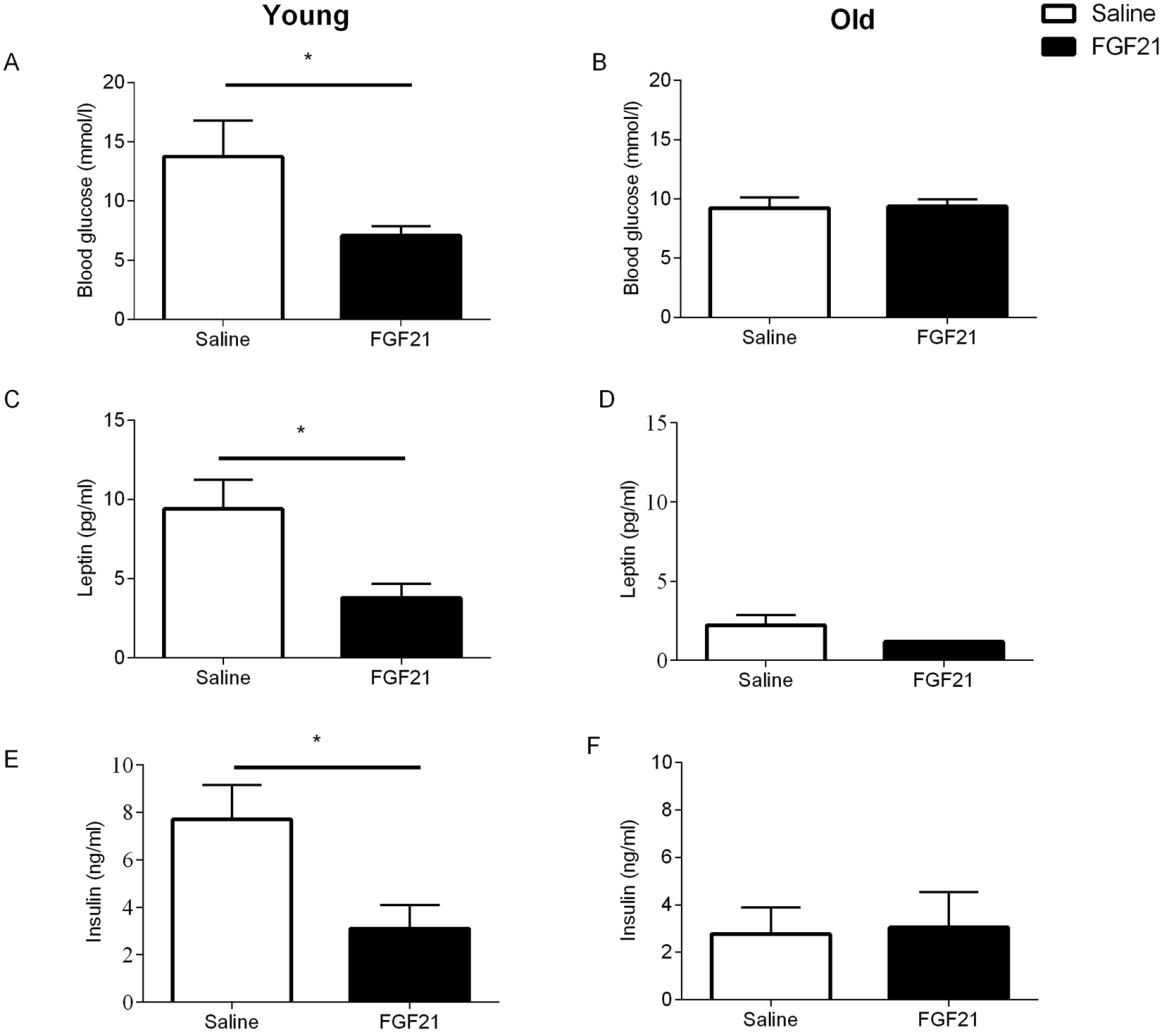
Treatment with FGF21 reduces blood glucose, and plasma leptin and insulin in the young Siberian hamster. Blood glucose (**A**), leptin (**B**) and insulin (**C**) of young and aged Siberian hamsters treated with vehicle (saline) or FGF21. Values are group mean ± SEM, n = 5–6 per treatment: *p < 0.05.

Energy expenditure was significantly increased by FGF21 treatment in both young and aged animals, with no difference between cohorts (Fig. 3A and B, p < 0.05). The increase in energy expenditure was not attributable to increased ambulatory activity as this was unaffected by age and/or treatment (Fig. 3C and D). RER was significantly reduced in the young cohort in response to FGF21 treatment (Fig. 3E, p < 0.05), whilst there was no significant effect in the aged cohort (Fig. 3F), suggesting that the aged cohort had an impaired ability to switch fuel/energy substrate utilisation in response to treatment when compared with the young cohort (p < 0.05). Interestingly, blood glucose concentration and plasma leptin and insulin concentrations were significantly reduced in the young cohort in response to FGF21 treatment (Fig. 4A,C and E respectively, p < 0.05). However, there was no effect of FGF21 treatment in the aged cohort (Fig. 4B,D and F).

### FGF21 induces central changes, but fails to induce beiging of white adipose tissue in aged animals

**Figure 5:**
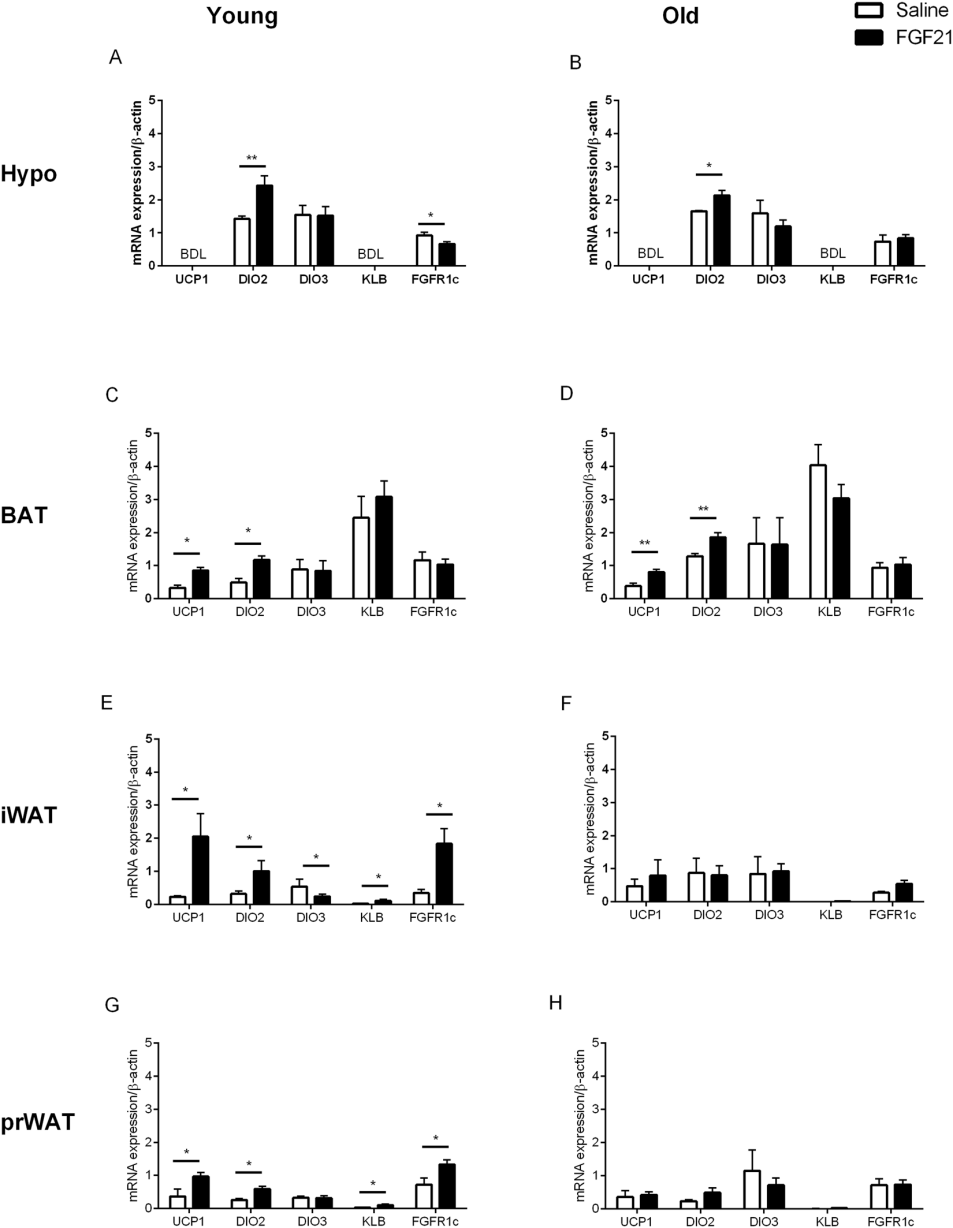
Treatment with FGF21 increases thermogenic genes in the hypothalamus and iBAT in young and aged Siberian hamsters, whilst the effects of treatment are lost in iWAT and prWAT in the aged Siberian hamster. Hypothalamus (**A** and **B**), iBAT (**C** and **D**), iWAT (**E** and **F**) and prWAT (**G** and **H**) gene expression profiles in young and aged Siberian hamsters treated with FGF21. Values are group mean ± SEM, n = 5–6 per treatment: *p < 0.05, **p < 0.01.

In the hypothalamus, Dio2 expression was increased in both young and aged cohorts in response to treatment (p < 0.05, Fig. 5A and B respectively). Interestingly, in the young cohort the FGFR1c was reduced in response to treatment (Fig. 5A p < 0.05). In iBAT from young and aged hamsters, treatment resulted in a significant increase in Ucp1 and Dio2 (Fig. 5C and D, p < 0.05). FGF21 treatment resulted in a significant increase in a plethora of genes (Ucp1, Dio2, KLB and FGFR1c) in iWAT (Fig. 5E, p < 0.05) and prWAT (Fig. 5G, p < 0.05) in the young cohort, but there was no effect of treatment in the aged cohort (Fig. 5F and H). In addition to these effects, treatment reduced DIO3 expression in iWAT in young cohort (Fig. 5F, p < 0.05).

### FGF21 reduces TAG, increased ERK phosphorylation and increased Atgl – an effect which is absent in aged animals

**Figure 6:**
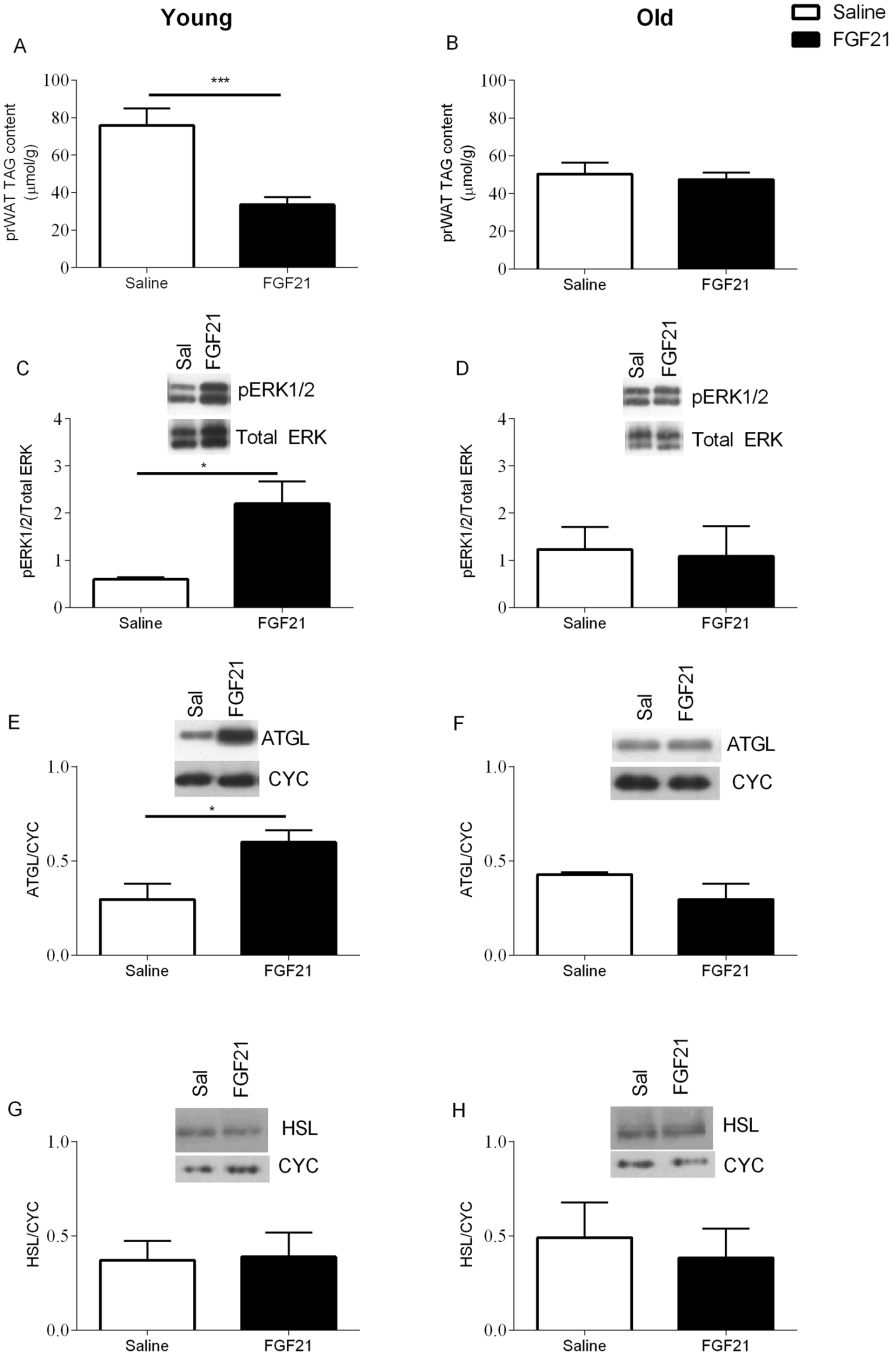
Treatment with FGF21 reduces prWAT TAG content and increases key phosphorylation events and AGTL in young Siberian hamsters. prWAT TAG content (**A** and **B**) of young and aged Siberian hamsters treated with FGF21. pERK1/2 and total ERK (**C** and **D**), ATGL (**E** and **F**) and HSL (**G** and **H**) content of prWAT. Values are group mean ± SEM, n = 5–6 per treatment: *p < 0.05 **p < 0.01.

Triglyceride (TAG) content in prWAT was significantly reduced by FGF21 treatment in the young cohort (Fig. 6A p < 0.05), whereas in the aged cohort TAG was unaffected by treatment (Fig. 6B). Interestingly, whilst key phosphorylation events downstream of the FGFR1c-KLB receptor complex were increased in response to treatment with FGF21 in the young cohort (extracellular signal-related kinases -pERK1/2), these were diminished in the aged cohort (Fig. 6C, p < 0.05, and 6D respectively). Furthermore, treatment resulted in a significant increase in the protein content of adipose triglyceride lipase (Atgl) in prWAT in the young cohort (Fig. 6E, p < 0.05), whilst there was no effect of treatment in the aged cohort (Fig. 6F). Interestingly there was no effect on treatment onhormone sensitive lipase (Hsl) (Fig. 6G and H respectively).

## Discussion

There are two major outcomes of our studies in Siberian hamsters. In young long day (LD) hamsters with increased adiposity we report that an FGF21 mimetic LY2405319 lowers body weight, adiposity and improves glycemia due to increased energy expenditure, in addition to a reduced daily caloric intake. Furthermore, similar to studies reported in mice, at the molecular level, these effects appear to be underpinned by recruitment of thermogenic pathways in brown and white adipose tissue. However, in the aged hamster, where adiposity is reduced to levels naturally found in the short-day (SD) “winter” photoperiod, despite maintenance of the LD “summer” photoperiod, we found that the body weight lowering effects of FGF21 were significantly attenuated. This is not a consequence of reduced receptor and/or co-factor expression in the hypothalamus or adipose tissue. However, key downstream phosphorylation events are lost in the aged cohort in response to treatment. Previously we demonstrated that the actions of FGF21 mimetics were diminished in SD animals^21, 22^. However, results were equivocal as to whether this was a consequence of the central photoperiodic state or a result of reduced adiposity. These findings in young versus aged animals, where adiposity is reduced despite the maintenance of the LD phenotype, support the view that adiposity is the key determinant regulating FGF21 responsiveness in the Siberian hamster. Furthermore, the studies support a central mechanism of action of FGF21; the observed hypophagia in response to treatment is apparent in young and aged animals, albeit with an attenuated response in the latter group. Importantly, this effect appears to be isolated to Siberian hamsters, as no effect has been shown in numerous studies of obese mice and rats^6, 21, 25^. Furthermore, in response to treatment with FGF21, hypothalamic DIO2, which governs the local availability of active thyroid hormone, is increased in young and aged animals, whilst FGFR1c is reduced in young animals in response to treatment suggesting a possible negative feedback loop^26^.

The hypophagia observed in the present study may be a reflection of the efficacy of FGF21 or a consequence of the metabolic compensation Siberian hamsters demonstrate after a period of fasting/starvation^27, 28^. A monoclonal antibody targeting the hypothalamic FGFR1 causes potent hypophagia in rodents and non-human primates^22, 29^. The reduced food intake demonstrated in these studies is associated with reduced meal duration and frequency in young animals, suggesting a possible effect on satiety and/or reward. Previously FGF21 has been shown to suppress sweet and alcohol preference, effects which are associated with decreased dopamine^17^. It has been previously shown that radiolabelled FGF21 crosses the blood-brain barrier, whilst *in situ* hybridisation studies have demonstrated FGFR1c expression in the hypothalamus of Siberian hamsters^22, 30^. Furthermore, KLB has been reported in discrete areas of the hypothalamus, deletion of which has been demonstrated to abrogate the metabolic effects of endogenous FGF21 administration^31^. However, in the current study, we were unable to detect KLB in the hypothalamus of Siberian hamsters. This may be a reflection of the sensitivity of the qPCR technique, but an alternative view is that the natural ligand for FGFR1c in the hypothalamus is locally produced FGF2 rather than peripheral FGF21. It has also been demonstrated that central FGF21 activates the sympathetic nervous system and intact sympathetic tone is required to mediate its effects at the periphery^31, 32^.

In the current study we observed a reduction in body weight in both young and aged Siberian hamsters treated with FGF21 that was associated with a reduction in food intake and an increase in energy expenditure. The reductions in body weight are a consequence of reduced subcutaneous and visceral adiposity. Whilst the increases in energy expenditure were not associated with an increase in ambulatory activity in either cohort. In the young cohort, FGF21 treatment was associated with a decrease in RER suggesting a shift in substrate utilisation from carbohydrate (COX) to fat (FOX) oxidation. These effects are broadly in line with observations in the genetic (ob/ob) or diet-induced mouse models of obesity^6^.

The inability of the aged cohort to utilise fat as an energy substrate correlates with the loss of induction of Ucp1 and Dio2 in interscapular and visceral WAT. It is therefore prudent to examine peripheral mechanisms that might explain the attenuation of responsiveness to FGF21, hence our analysis of actions of FGF21 in promoting fat oxidation. The two predominant lipases in WAT are Hsl and Atgl, the latter of which favours TAG substrates and is the rate-determining enzyme for lipolysis in adipose tissue^33, 34^. Increased mRNA and protein expression of Hsl and Atgl have previously been reported in transgenic mice that overexpress FGF21^35^. However, treatment of 3T3-L1 adipocytes with FGF21 did not increase activity of either enzyme, suggesting an indirect effect^14, 36, 37^. Here we demonstrate, in addition to the effects on RER, there is a significant reduction in prWAT wet weight and TAG content in young Siberian hamsters treated with FGF21. Furthermore, there is an increase in the protein level of Atgl in this tissue, indicative of increased lipolysis. In addition, key downstream signalling pathways (ERK1/2 phosphorylation) are activated in this tissue in response to treatment, however this response is lost in the aged cohort, suggesting age-related FGF21 resistance, which warrants further investigation. The current experimental design does not discriminate whether this is a direct action of FGF21 or a consequence of the FGF21-induced reduction in food intake, so further studies are required to resolve this. However, given that food intake is significantly reduced by FGF21 in the aged cohort (albeit attenuated in comparison to the young animals treated with FGF21), but TAG content and Atgl abundance are not affected in the aged cohort, the difference in response may well reflect changes within adipose tissue. The changes in gene expression are also consistent with direct actions of FGF21 in adipose tissue. We observed that both brown and white adipose tissue in hamsters express both FGFR1c and KLB. Targeted knockout of these receptors from adipocytes greatly reduces the responsiveness of mice to FGF21, and conversely over-expression of KLB in this tissue increases sensitivity to FGF21^38^. Pharmacological doses of FGF21 were shown to induce a thermogenic gene response in both BAT and thermogenically competent WAT depots. This was associated with a large induction of Pgc-1α protein, whilst FGF21 is necessary for the cold-induced recruitment of brown-like (beige) adipocytes in iWAT^2, 39^. Firstly, in the present study in the young and aged Siberian hamster, treatment with FGF21 increased Ucp1 and Dio2 in iBAT. This suggests an increase in thermogenic activity. Interestingly, energy expenditure is increased in both young and aged animals treated with FGF21. Our data therefore suggests increases in thermogenic gene expression in iBAT is sufficient to drive increased energy expenditure in this species in response to treatment. Secondly, in iWAT and prWAT, Ucp1, Dio2, KLB and FGFR1c are increased in response to treatment, suggesting an increase in thermogenic capacity and the recruitment of brown-like (beige) adipocytes, in young animals. These effects, however, where diminished in the aged cohort of animals. Interestingly, these animals lack the ability to utilise fat as an energy substrate in response to treatment. An inability to ‘beige’ WAT suggests a metabolic inflexibility in the aged cohort, which possibly attenuates the response to treatment. Interestingly, the importance of FOX in cold-induced thermogenesis was recently demonstrated. Adipose specific knockout of carnitine palmitoyltransferase 2 (Cpt2), an obligate step in mitochondrial long chain FOX, renders mice hypothermic after an acute cold challenge^40^. Whilst the induction of KLB and FGFR1c suggest that FGF21 plays an important role in thermogenically competent WAT depots.

Interestingly blood glucose, and plasma leptin and insulin were reduced by FGF21 treatment in the young cohort, but these effects were attenuated in the aged cohort. Previously we had shown that treatment with FGF21 did not affect fasting plasma glucose concentrations, however, plasma insulin and leptin concentrations were significantly reduced by treatment, effects which were attenuated in SD animals^21^. This demonstrates a possible role for FGF21 in non-fasted (i.e. fed) animals, and once again the importance of metabolically active adipose tissue for FGF21 responsiveness.

Metabolic syndrome represents a significant burden to public health and the endocrine family of FGFs have emerged as novel regulators of glucose and lipid metabolism^41^. The beneficial effects of FGF21 on body weight, food intake and energy expenditure have resulted in substantial interest in FGF21 as a potential treatment for obesity and metabolic syndrome. Our data support a clear physiological mechanism of action for FGF21 in regulating FOX via its action in metabolically active WAT depots, via increasing Atgl and reducing TAG, and increasing the transcription of thermogenic genes. It is tempting to suggest the thermogenic effects of FGF21 on adipose tissue may underlie many of its beneficial effects observed *in vivo*. Given that human ageing is associated with increased risk of obesity and metabolic syndrome, our study highlights the need for further research into FGF21 mediated improvements in metabolically inflexible adipose tissue. Furthermore the data supports the hypothesis that adiposity is the key determinant in regulating FGF21 responsiveness in the Siberian hamster rather than the central photoperiodic state.

## Methods

### Animals

Adult male Siberian hamsters (Phodopus sungorus) comprising a young cohort [3 months] and an aged cohort [18 months, as previously defined (Horton and Yellon, 2001; McKeon, 2011)] were obtained from a colony maintained at the University of Nottingham Biomedical Services Unit^42^. All studies were approved by the University of Nottingham Local Ethical Review Committee and carried out in accordance with the UK Animals (Scientific Procedures) Act of 1986 (project license: PPL 40/3604). Siberian hamsters were group housed and maintained at 21 °C and 40% humidity, and were allowed ad libitum access to food (Teklad 2019, Harlan, UK) and water. Animals were housed from birth in LD condition of 16 hours light: 8 hours dark with lights out at 11:00 GMT.

### FGF21 ﻿(LY2405319) treatment

Prior to surgery animals were singly housed to accurately determine food intake. Young (n = 6 per treatment, mean 37.9 ± 1.6 g) and aged Siberian hamsters (n = 5 per treatment, mean 31.1 ± 2.0 g) received a subcutaneously (s.c.) implanted Alzet osmotic mini-pumps (Model 1007D, Charles River) releasing vehicle (saline) or LY (FGF21; 3 mg/kg/day) for 7 days. Mini-pumps were inserted below the skin on the flank of the Siberian hamster under 1.5% isoflurane anaesthesia. Hamsters were treated with analgesic (5 mg/kg s.c., maintained for 3 days with additional fluids, 0.5 ml/day, Rimadyl, Pfizer, Kent, UK) and the wound closed with Michel clips. Body weight and food intake were recorded daily, shortly before lights out. Three days post-surgery the animals were transferred to metabolic cages for a 48 hrs, with the first 24 hrs used as the habituation period and the final 24 hrs for the measurement of the metabolic parameters described below.

### Metabolic cages

Oxygen consumption (VO_2_) and carbon dioxide production (VCO_2_) were measured concurrently using a modified open-circuit calorimeter known as comprehensive laboratory animal monitoring system (CLAMS) as previously described^43^. VO_2_ and VCO_2_ were then used to calculate energy expenditure (EE) and respiratory exchange ratio (RER) as previously described^44, 45^. Measurements were taken at 9 minute intervals for 24 hrs.

### Assessment of body composition

Siberian hamsters were imaged by X-ray computed tomography (CT) scanning pre- and post-treatment. Briefly, CT imaging of the Siberian hamster under general anaesthesia (1.5% isoflurane) was carried out using the CT subsystem (tube settings at 35 kVp, 0.61 mA and exposure time 170 ms) on a nanoPET-CT scanner (Medisoc Medical Imaging Systems, Budapest, Hungary). To quantify the volume of adiposity, subcutaneous and visceral fat depots were identified via connected and global thresholding (200–500 and 200–350 Hounsfield units (HU) respectively) using VivoQuant multi-modal image processing software (inviCRO, Boston, USA).

### Molecular analysis

Animals were euthanised via an i.p. injection of sodium pentobarbitone (Euthatal, Rhone Merieux, Harlow, UK) and samples of the brain, liver, interscapular BAT (iBAT) and interscapular and perirenal WAT (iWAT and prWAT respectively) were collected, snap frozen on dry ice and stored at −80 °C until required. Total RNA was extracted from 20 mg of frozen wet tissue using TRIzol reagent (Invitrogen). The hypothalamus was dissected from the brain as previous described^46^. Aliquots of RNA were assessed for purity and quantified via Nanodrop ND-100 (Thermo Fisher Scientific, Wilmington, USA). Reverse transcription was carried out using 500 ng of total RNA using the SuperScript III cDNA kit (Invitrogen). Taqman primers and probes sets were obtained from Applied Biosystems (see Table 1) using the Siberian hamster sequence and assembly (Accession: PRJNA318271 ID: 318271). Real-time PCR was performed using PCR Universal Master Mix (Applied Biosystems) in a Micro-Amp 96-well plate using an ABI Prism 7000 Sequence Detection System (Applied Biosystems). Assays were performed in triplicate. The threshold (Ct) values for each triplicate were averaged and the quantification of expression of each gene relative to β-actin determined using the standard curve method^47^.

**Table 1:** Primers and probes.

Gene	Forward primer	Reverse primer	Probe sequence
Β-actin	CGTGCGTGACATCAAAGAGAA	AGCAGTGGCCATCTCTTGCT	CTGTGCTACGTTGCCCT
Dio2	ACCACCACCTTCCTTTGCAA	GCGGAAGGCTGGCAGTT	AAGCAGAGTGCCCAGGA
Dio3	GTGCATCCGCAAGCATTTC	ACTTCAGGCTCGGGATGGT	TGCGCCGTCGCCA
FGFR1c	GGAGTTCATGTGTAAGGTGTACAGTGA	GTGGTATTAACTCCAGCAGTCTTCAG	CCTCAXATCCAGTGGCTGAAGCACATT
KLB	CTGATCAAGGCACATTCGAAAG	TGGGACCCCAAGGTGATG	ACGACAAAAACTTCCGCCCTCATCAGAAG
Ucp1	CCGGCTTCAGATCCAAGGT	TCGGCAACCCTTCTGTTTTT	TGTCCTTGGGACCATCACCACCCT

### Circulating metabolites and hormones

Blood samples collected in EDTA tubes on ice via cardiac puncture, blood glucose measured (HemoCue 201 system, Angelholm, Sweden) and plasma collected post-centrifugation and stored at −80 °C until required. ELISA kits (Millipore, MA, USA) were used to measure circulating levels of FGF21(rat/mouse kit EZRMFGF21-26 ﻿K), leptin (mouse kit EZML-82 K), insulin (rat/mouse kit EZRMI-13 K) in plasma samples.

### Triglyceride and protein determination

Triglyceride (TAG) content of perirenal white adipose tissue (prWAT) was performed as previously described^45^. Proteins were extracted using a HEPES Lysis buffer and quantified by densitometry (Aida Image Analyser, version 4.27). Western blotting was performed and resulting membranes were probed with primary antibodies to adipose triglyceride lipase (ATGL, Abcam, #ab109251), hormone sensitive lipase (HSL, NEB, #4017) pERK1/2 (ERK, Cell Signalling, #9107), total ERK (Cell Signalling, #4370) (both bands) and cyclophilin B (CYC, Abcam, #ab74173). Membranes were blocked for 1 hour at room temperature and then incubated at 4 °C overnight in primary antibody dilutions (ATGL and CYC 1 in 1000; HSL 1 in 500; ERK1/2 1 in 500; total ERK 1 in 1000). Blots were subsequently washed in TBS-Tween (0.1% w/v) and incubated at room temperature for 1 hour in secondary antibody at a dilution of 1 in 2000. ATGL and ERK blocking and antibody dilutions were performed in TBS-Tween containing 5% w/v non-fat dried milk. Blocking and antibody dilutions for HSL and CYC were performed in TBS-Tween containing 5% BSA. Detection of membranes was performed using Amersham ECL Prime Western Blotting Detection Reagent (cat. #RPN2232) and Amersham Hyperfilm ECL (ref: 28906836).

### Statistical analysis

Descriptive statistics (mean ± S.E.M.) were generated using Graphpad Prism (Prism 6.0, GraphPad, San Diego, CA, USA). After checking for normality of distribution and equality of variance, body weight, food intake CLAMS data were analysed using a two-way (treatment × sampling time) repeated measures ANOVA, with Bonferroni corrected post-hoc T-tests when a significant main effect or interaction was detected. Tissue weights and metabolite analysis were analysed by students unpaired T-test, and gene expression analysis were analysed using one-way ANOVA. No animals were excluded from the analysis. Statistical significance was declared at p < 0.05.

## Electronic supplementary material

Supplementary Information
