# Supplementary material for: Reduced adiposity attenuates FGF21 mediated metabolic improvements in the Siberian hamster
Source: Sci Rep. 2017 Jun 26;7:4238. doi: 10.1038/s41598-017-03607-x (PMC5484705; doi:10.1038/s41598-017-03607-x)
Supplement: Supplementary file 1 — Supplementary Information [file 41598_2017_3607_MOESM1_ESM.pdf]

Reduced adiposity attenuates FGF21 mediated metabolic improvements in the Siberian hamster

Jo E Lewis[1*], Ricardo J Samms[2], Scott Cooper[1], Jeni C Luckett[3], Alan C Perkins[3], Andrew C Adams[2], Kostas Tsintzas[4], Francis J P Ebling[1]

[1]School of Life Sciences, University of Nottingham Medical School, Queen's Medical Centre, Nottingham NG7 2UH, UK

[2]Lilly Research Laboratories, Indianapolis, IN 46285, USA

[3]Radiological Sciences, School of Medicine, University of Nottingham Medical School, Queen's Medical Centre, Nottingham NG7 2UH, UK

[4]MRC/ARUK Centre for Musculoskeletal Ageing, School of Life Sciences, University of Nottingham Medical School, Queen's Medical Centre, Nottingham NG7 2UH, UK

Supplementary Figure 1: Ageing is associated with reduced adiposity in the Siberian hamster. Body weight (A), average daily food intake (B), representative images of young and aged Siberian hamsters (C), energy expenditure (D), ambulatory activity (E), RER (F), plasma FGF21 (G), leptin (H) and insulin (I) of young and aged Siberian hamsters. Values are group mean ± SEM n = 5-6 per group * $p < 0.05$ ** $p < 0.01$ ***.

Supplementary Figure 2: FGFR1c and KLB expression in the hypothalamus and adipose tissue of young and aged Siberian hamsters. Values are group mean ± SEM n = 5-6 per group.

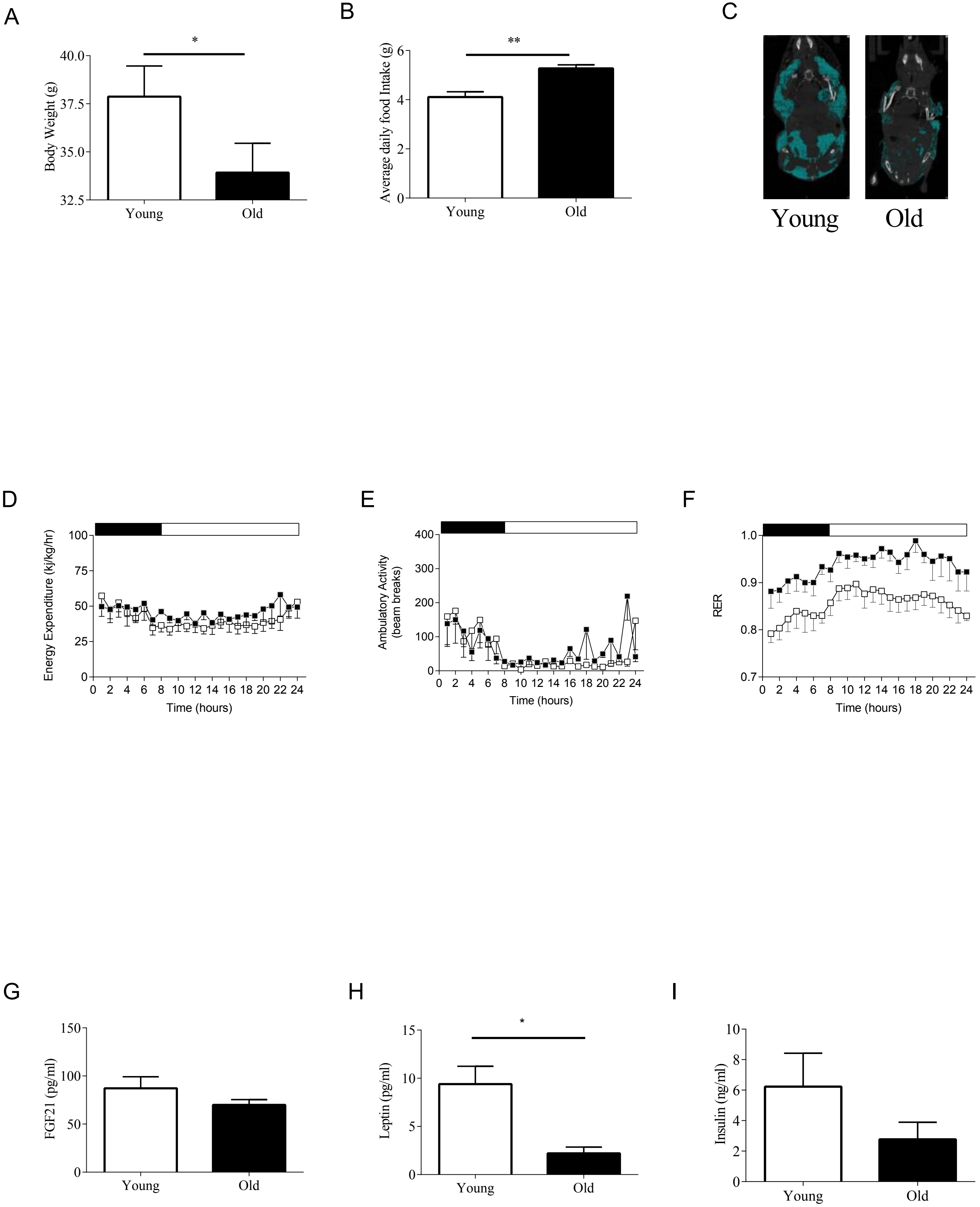
A
*
Body Weight (g)
40.0
37.5
35.0
32.5
Young
Old
B
**
Average daily food Intake (g)
6
4
2
0
Young
Old
C
Young
Old
D
Energy Expenditure (kj/kg/hr)
100
75
50
25
0
0 2 4 6 8 10 12 14 16 18 20 22 24
Time (hours)
E
Ambulatory Activity (beam breaks)
400
300
200
100
0
0 2 4 6 8 10 12 14 16 18 20 22 24
Time (hours)
F
RER
1.0
0.9
0.8
0.7
0 2 4 6 8 10 12 14 16 18 20 22 24
Time (hours)
G
FGF21 (pg/ml)
150
100
50
0
Young
Old
H
*
Leptin (pg/ml)
15
10
5
0
Young
Old
I
Insulin (ng/ml)
10
8
6
4
2
0
Young
Old

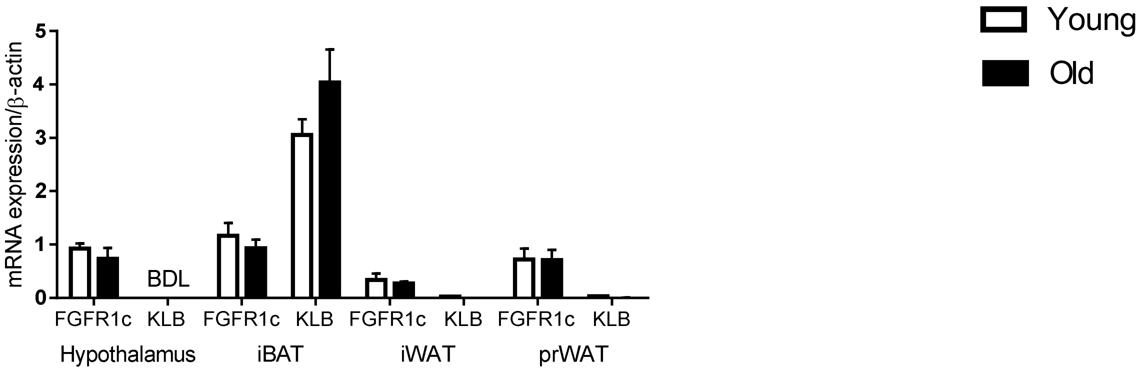

mRNA expression/β-actin
5
4
3
2
1
0
BDL
FGFR1c
KLB
FGFR1c
KLB
FGFR1c
KLB
FGFR1c
KLB
Hypothalamus
iBAT
iWAT
prWAT
Young
Old
